# P02-006 - A novel PSTPIP1 mutation in PAPA syndrome

**DOI:** 10.1186/1546-0096-11-S1-A113

**Published:** 2013-11-08

**Authors:** B Fathalla, M Al-Mutawa, F Al-Amri, S Al-Dosari, M Kambouris, H El-Shanti

**Affiliations:** 1 Hamad Medical Corporation, Doha, Qatar; 2 Shafallah Medical Genetics Center, Doha, Qatar

## Introduction

Pyogenic arthritis, pyoderma gangrenosum, and acne (PAPA) syndrome is an autosomal dominant autoinflammatory disease caused by mutations in the proline-serine-threonine phosphatase-interacting protein 1, *PSTPIP1*.

The produced protein is a cytoskeleton-associated adaptor protein that modulates T-cell activation, cytoskeletal organization and IL-1β release.

The only two mutations described so far, A230T and E250Q, have been found in patients and families, and are thought to disrupt the binding of PSTPIP1 with PTP-PEST, a regulatory phosphatase, and increase its avidity for pyrin in the cytosol, thereby dysregulating IL-1β production. PAPA syndrome typically presents with recurrent sterile, erosive arthritis in childhood, resulting in significant joint destruction. By puberty, joint problems tend to subside and cutaneous symptoms increase including pathergy, frequently with abscesses at the sites of injections, severe cystic acne, and recurrent non-healing sterile ulcers, often diagnosed as pyoderma gangrenosum.

## Case report

We describe a a four year old Jordanian male, born to healthy non-consanguineous parent, who presented with cutaneous abscesses at the age of 6 months and then at 18 months at the vaccination injection sites. At the age of 20 months he developed cellulitis. At the age of 23 months, he had acute arthritis of the right ankle. He developed acute arthritis of the left wrist at 24 months, and the right wrist at 27 months and then the right elbow at the age of 45 months. He has two older sisters and the family history is negative for similar conditions. The sequencing of the coding region of *PSTPIP1* and flanking intronic regions revealed a *de novo* variation p.Asp246Asn (p.D246N) in the child. The variant is predicted to be probably damaging by Polyphen and was not found in 360 ethnically-matched control chromosomes.

## Discussion

We describe a 4 year old Jordanian boy with a typical clinical presentation of PAPA syndrome, with the exception of the absence of pyoderma gangrenosum and acne. However, both these findings may occur later in the course of the disease, mostly after puberty. We anticipate that this variation is the mutation the explains the symptoms in this child since it falls within the coiled coil domain that harbors all the previously described mutations. Since the E250Q and A230T variants of *PSTPIP1* were shown to severely abrogate binding to PTP-PEST in yeast two hybrid and co-immunoprecipitation experiments, we anticipate this mutation does the same.

## Disclosure of interest

None declared.
